# Isolation and analyses of lamina propria lymphocytes from mouse intestines

**DOI:** 10.1016/j.xpro.2022.101366

**Published:** 2022-05-06

**Authors:** Eunha Kim, Melissa Tran, Yanyi Sun, Jun R. Huh

**Affiliations:** 1 Department of Immunology, Blavatnik Institute, Harvard Medical School, Boston, MA 02115, USA; 2 Department of Biological Chemistry and Molecular Pharmacology, Blavatnik Institute, Harvard Medical School, Boston, MA 02115, USA; 3 Evergrande Center for Immunologic Diseases, Harvard Medical School and Brigham and Women’s Hospital, Boston, MA 02115, USA

**Keywords:** Cell isolation, Single Cell, Flow Cytometry/Mass Cytometry, Immunology

## Abstract

Investigating intestinal immune responses is critical to understanding local and systemic immunity. However, obtaining resident intestinal immune cells with high cell viability can be challenging. Here, we provide an optimized protocol to isolate lamina propria lymphocytes from the small and large intestines, including lymphocyte activation for cytokine expression analysis and techniques for surface and intracellular antibody staining and flow cytometry. This protocol can be used for isolating and analyzing tissue-resident immune cells from other tissues with specified modifications.

For complete details on the use and execution of this protocol, please refer to Kim et al. (2022).

## Highlights

• Optimized protocol to isolate and analyze mouse intestinal lamina propria lymphocytes
• Stimulation of isolated cells for intracellular cytokine analysis
• Assessment of T and B cell subtype composition via antibody staining and flow cytometry

## Graphical abstract

**Figure:**
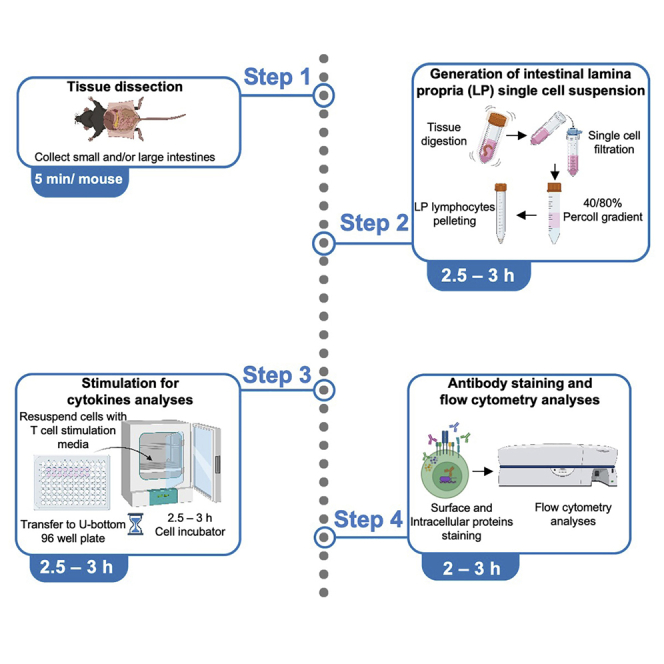

## Before you begin

### Institutional permission for animal experiment

All experiments were conducted in accordance with procedures approved by the Institutional Animal Care and Use Committee of Harvard University, Boston, USA.

The protocol below describes the specific steps for isolating and analyzing intestinal lamina propria lymphoid cells.

***Alternatives:*** This protocol is optimized for intestinal lamina propria immune cells. However, we have also used this protocol for isolating and analyzing tissue-resident immune cells from other tissues (e.g., placenta, liver, brain, meninges), with slight modifications. For example, if working with non-mucosal tissues, EDTA and DTT digestion steps can be skipped and Liberase concentration can be adjusted to 50 μg/mL.

Gut epithelial cells and intraepithelial lymphocytes can also be harvested from this protocol; see note after step 10 for more details.

### Mice

The composition of immune cells in the intestinal lamina propria is greatly affected by the gut microbiota (Round and Mazmanian, 2009), as well as sex, age, and strain (Elderman et al., 2018; Man et al., 2014). Therefore, the use of vivarium-, age-, sex-, strain-matched control and experimental groups is required. Animals were purchased from the same barrier and vendor for each experiment. All wild-type (WT) conventional 8–12 weeks old C57BL/6 male or female mice were purchased from Taconic Biosciences (USA).

### Prepare buffers and media

**Timing: 0.5 h**

1. Prepare the necessary buffers and media before starting the experiment. Recipes and storage conditions for the buffers and media can be found in the materials and equipment section.

### Antibody panel preparation

**Timing: 0.25–0.5 h**

2. Prepare two flow cytometry antibody panels for surface marker proteins (Table 1) and intracellular cytokines/transcription factors (Table 2). Antibodies need to be prepared immediately before the staining step as a master mixture. Detailed staining panel information and dilution factors can be found in the materials and equipment section. HBSS will be used for surface marker proteins antibodies mixture, and 1× permeabilization buffer will be used for intracellular cytokines/transcription factors master mixture.

**Table 1:** Surface markers staining panel

Fluorophore	Marker	Clone	Dilution factor
APC-Cy7	CD45	30-F11	1:400
Brilliant Blue 700	CD4	RM4-5	1:200
Alexa Fluor 700	TCRβ	H57-597	1:200
Brilliant Violet 605	CD8a	53-6.7	1:200
Brilliant Violet 605	CD19	6D5	1:200
PE-Cy7	CD3ε	145-2C11	1:200

**Table 2:** Intracellular cytokines/transcription factors staining panel

Fluorophore	Marker	Clone	Dilution factor
Alexa Fluor 488	IL-17A	TC11-18H10.1	1:200
Brilliant violet 421	IFN-γ	XMG1.2	1:200
APC	FoxP3	FJK-16s	1:200
PE	RORγt	B2D	1:200

***Note:*** We used the same fluorophore for CD8a and CD19 because we can differentiate CD8 T cells by gating on TCRβ, CD3ε-positive and CD8a (BV605), and B cells on TCRβ, CD3ε-negative, and CD19-positive (BV605). However, different fluorophores can be chosen for either CD8a or CD19 if it is possible to add more fluorophores.

## Key resources table

**Table:** 

REAGENT or RESOURCE	SOURCE	IDENTIFIER
**Antibodies**		

Anti-mouse CD45 (Clone ID 30-F11) APC-Cy7	BD Biosciences	Cat# 557659; RRID: AB_396774
Anti-mouse CD4 (Clone ID RM4-5) BB700	BD Biosciences	Cat# 566407; RRID: AB_2744427
Anti-mouse TCRβ (Clone ID H57-597) A700	BioLegend	Cat# 109224; RRID: AB_1027648
Anti-mouse CD8a (Clone ID 53-6.7) BV605	BioLegend	Cat# 100744; RRID: AB_2562609
Anti-mouse CD19 (Clone ID 6D5) BV605	BioLegend	Cat# 115540; RRID: AB_2563067
Anti-mouse IL-17A (Clone ID TC11-18H10.1) A488	BioLegend	Cat# 506910; RRID: AB_536012
Anti-mouse IFN-γ (Clone ID XMG1.2) BV421	BioLegend	Cat# 505830; RRID: AB_2563105
Anti-mouse RORγt (Clone ID B2D) PE	Thermo Fisher Scientific	Cat# 12-6981-82; RRID: AB_10807092
Anti-mouse FoxP3 (Clone ID FJK-16s) APC	Thermo Fisher Scientific	Cat# 17-5773-82; RRID: AB_2573254
Anti-mouse CD3ε (Clone ID 145-2C11) PE-Cy7	Thermo Fisher Scientific	Cat# 25-0031-82; RRID: AB_469572
Rat anti-mouse CD16/CD32 (Mouse BD Fc Block^TM^) (Clone ID 2.4G2)	BD Biosciences	Cat#553142; RRID: AB_394656

**Chemicals, peptides, and recombinant proteins**		

Liberase^TM^ TM research grade	MilliporeSigma	Cat# 5401127011
DNase I	MilliporeSigma	Cat# 10104159001
Phorbol 12-myristate 13-acetate (PMA)	Sigma-Aldrich	Cat# P1585
Ionomycin	Sigma-Aldrich	Cat# I0634
GolgiPlug	BD Biosciences	Cat# 555029
Dithiothreitol (DTT)	MilliporeSigma	Cat# 10708984001
Ethylenediaminetetraacetic acid (EDTA) 0.5M pH 8.0	Corning	Cat# 46-034-Cl
Fetal bovine serum (FBS)	HyClone	Cat# SH30910.03
FBS	Corning	Cat# 35-010-CV
Percoll	MilliporeSigma	Cat# GE17-0891-01
RPMI-1640 1×, w/o L-glutamine	Corning	Cat# 15-040-CV
GlutaMAX^TM^-I (100×)	Thermo Fisher Scientific	Cat# 35050-061
Gentamicin Sulfate	Corning	Cat# 30-005-CR
2-Mercaptoethanol	Thermo Fisher Scientific	Cat# 21985-023
Penicillin Streptomycin solution, 100×	Corning	Cat# 30-002-Cl
Bovine Serum Albumin, fraction V	MP Biomedicals	Cat# MFCD00130384
10× Hank’s Balanced Salt Solution (HBSS)	Thermo Fisher Scientific	Cat# 14065056
1× HBSS (without calcium, magnesium, phenol red)	Corning	Cat# 21-022-CM
Molecular biology grade water	Corning	Cat# 46-000-CV

**Critical commercial assays**		

Foxp3/ Transcription Factor Staining Buffer Kit	Thermo Fisher Scientific	Cat# 00-5523-00
LIVE/DEAD Fixable Aqua Dead Cell stain kit	Thermo Fisher Scientific	Cat# L34966

**Experimental models: Organisms/strains**		

C57BL/6	Taconic	Cat# B6 (C57BL/6NTac)

**Software and algorithms**		

FlowJo 10	BD Biosciences	https://www.flowjo.com

**Other**		

BD LSR II Flow Cytometer	BD Biosciences	N/A
96 well U-bottom plate	Corning	Cat# 353077
Square petri dish with grid	Fisher Scientific	Cat# 07-757-11A
100 um Strainer	VWR	Cat# 89858-040
UltraComp eBeads™ Compensation Beads	Thermo Fisher Scientific	Cat# 01-2222-42
ArC™ Amine Reactive Compensation Bead Kit	Thermo Fisher Scientific	Cat# A-10346
Falcon™ Plastic Disposable Transfer Pipets	Fisher Scientific	Cat# 13680-50
Disposable Borosilicate Glass Pasteur Pipets	Fisher Scientific	Cat# 13-678-20D

## Materials and equipment

**Table:** Liberase stock

Reagent	Final concentration	Amount
RPMI-1640	n/a	20 mL
Liberase™ TM Research Grade (100 mg)	5 mg/mL	n/a
**Total**	**n/a**	**20 mL**

***Note:*** Reconstitute the entire vial and aliquot the Liberase stock into 1.5 mL microcentrifuge tubes (1 mL/tube). Gently agitate the vial at 4°C until the enzyme is completely dissolved (∼30 min). The stock can be stored at −20 to −80°C.

**Table:** DNase I stock

Reagent	Final concentration	Amount
HBSS (w/o Ca^2+^, Mg^2+^)	n/a	20 mL
DNase I (100 mg)	5 mg/mL	n/a
**Total**	**n/a**	**20 mL**

***Note:*** Reconstitute the entire vial and aliquot the DNase I stock into 1.5 mL microcentrifuge tubes (1 mL/tube). Gently agitate the vial at 4°C until the enzyme is completely dissolved (∼30 min). Do not vortex to dissolve. The stock can be stored at −20 to −80°C.

**Table:** EDTA-DTT buffer

Reagent	Final concentration	Amount
HBSS (w/o Ca^2+^, Mg^2+^)	n/a	14.25 mL/sample
0.5 M EDTA	1 mM	0.3 mL/sample
1 M DTT	1 mM	0.15 mL/sample
FBS (Corning)	2%	0.3 mL/sample
**Total**	**n/a**	**15 mL/sample**

***Note:*** Prepare and use on the day of experiment. Prewarm HBSS at 37°C before use.

**Table:** Digestion solution

Reagent	Final concentration	Amount
RPMI-1640	n/a	4.82 mL/sample (for SI), 4.8 mL/sample (for LI)
5 mg/mL Liberase stock	31.25 μg/mL (for SI), 62.5 μg/mL (for LI)	31.25 μL/sample (for SI), 62.5 μL/sample (for LI)
5 mg/mL DNase I stock	50 μg/mL	50 μL/sample
FBS (Corning)	2%	0.1 mL/sample
**Total**	**n/a**	**5 mL/sample**

***Note:*** Prepare and use on the day of experiment. Prewarm RPMI-1640 at 37°C before use.

**Table:** 80% Percoll solution

Reagent	Final concentration	Amount
Percoll	80%	2 mL/sample
10× HBSS	1×	0.25 mL/sample
Molecular grade water	n/a	0.25 mL/sample
**Total**	**n/a**	**2.5 mL/sample**

***Note:*** Prepare and use on the day of experiment. Equilibrate to 20°C–22°C before use. The addition of HBSS to Percoll is required to make Percoll isotonic with physiological conditions and maintain osmotic pressure in cells.

**Table:** 40% Percoll solution

Reagent	Final concentration	Amount
80% Percoll	40%	2.5 mL/sample
RPMI-1640	n/a	2.4 mL/sample
FBS (Corning)	2%	0.1 mL/sample
**Total**	**n/a**	**5 mL/sample**

***Note:*** Prepare and use on the day of experiment. Equilibrate to 20°C–22°C before use.

**Table:** FACS buffer

Reagent	Final concentration	Amount
HBSS (w/o Ca^2+^, Mg^2+^)	n/a	500 mL
BSA	0.5%	2.5 g
0.5 M EDTA	2 mM	2 mL
Penicillin/Streptomycin (100×)	1×	5 mL
**Total**	**n/a**	**507 mL**

***Note:*** Can be prepared in advance and stored at 4°C for at least 4 months. Filtration through a 0.2 um vacuum filter is recommended.

**Table:** T cell culture media

Reagent	Final concentration	Amount
RPMI-1640	n/a	434 mL
FBS (Hyclone)	10%	50 mL
Gentamicin sulfate (50 mg/mL)	50 μg/mL	0.5 mL
GlutaMAX 100×	2×	10 mL
2-mercaptoethanol (55 mM)	55 μM	0.5 mL
Penicillin/Streptomycin (100×)	1×	5 mL
**Total**	**n/a**	**500 mL**

***Note:*** Can be prepared in advance and stored at 4°C for up to 1 month.

**Table:** 2× T cell stimulation media (Enough for 50 samples)

Reagent	Final concentration	Amount
T cell culture media	n/a	5 mL
PMA (1 mg/mL)	100 ng/ mL	0.5 μL
Ionomycin (2 mM)	2 μM	5 μL
GolgiPlug (1 mg/mL)	2 μg/mL	10 μL
**Total**	**n/a**	**5.0155 mL**

***Note:*** Prepare and use on the day of experiment. Prewarm at 37°C before use.

**Table:** FoxP3 fixation/ permeabilization working solution (Enough for 25 samples)

Reagent	Final concentration	Amount
FoxP3 Fixation/Permeabilization concentrate	25%	1 mL
FoxP3 Fixation/Permeabilization diluent	75%	3 mL
**Total**	**n/a**	**4 mL**

***Note:*** Freshly prepare before use.

**Table:** 1× Permeabilization buffer (Enough for 30 samples)

Reagent	Final concentration	Amount
10× Permeabilization buffer	1×	2 mL
Distilled water	n/a	18 mL
**Total**	**n/a**	**20 mL**

***Note:*** Freshly prepare before use.

**Table:** Fixable live/dead viability staining solution (Enough for 20 samples)

Reagent	Final concentration	Amount
LIVE/DEAD Fixable Aqua Dead Cell stain kit	500×	2 μL
HBSS (w/o Ca^2+^, Mg^2+^)	n/a	1 mL
**Total**	**n/a**	**1.002 mL**

***Note:*** Freshly prepare before use.

**Table:** Fc Blocker solution (Enough for 20 samples)

Reagent	Final concentration	Amount
Rat anti-mouse CD16/CD32 (Mouse BD Fc Block^TM^) (Clone ID 2.4G2)	1 μg/mL	2 μL
HBSS (w/o Ca^2+^, Mg^2+^)	n/a	1 mL
**Total**	**n/a**	**1.002 mL**

***Note:*** Freshly prepare before use.

**Table:** Surface antibody staining solution (Enough for 20 samples)

Reagent	Final concentration	Amount
CD45 (APC-Cy7)	1 μg/mL	2.5 μL (1:400 dilution)
CD4 (BB700)	1 μg/mL	5 μL (1:200 dilution)
TCRβ (A700)	2.5 μg/mL	5 μL (1:200 dilution)
CD8a (BV605)	1 μg/mL	5 μL (1:200 dilution)
CD19 (BV605)	1 μg/mL	5 μL (1:200 dilution)
CD3ε (PE-Cy7)	1 μg/mL	5 μL (1:200 dilution)
HBSS (w/o Ca^2+^, Mg^2+^)	n/a	1 mL
**Total**	**n/a**	**1.0275 mL**

***Note:*** Freshly prepare before use.

**Table:** Intracellular antibody staining solution (Enough for 20 samples)

Reagent	Final concentration	Amount
IL-17A	2.5 μg/mL	5 μL (1:200 dilution)
IFN-γ	1 μg/mL	5 μL (1:200 dilution)
FoxP3	1 μg/mL	5 μL (1:200 dilution)
RORγt	1 μg/mL	5 μL (1:200 dilution)
1× Permeabilization buffer	n/a	1 mL
**Total**	**n/a**	**1.02 mL**

***Note:*** Freshly prepare before use.

## Step-by-step method details

### Intestinal tissue collection

**Timing: 5 min/ mouse**

These steps collect fresh intestinal tissues to isolate lymphocytes.

**Figure 1:**
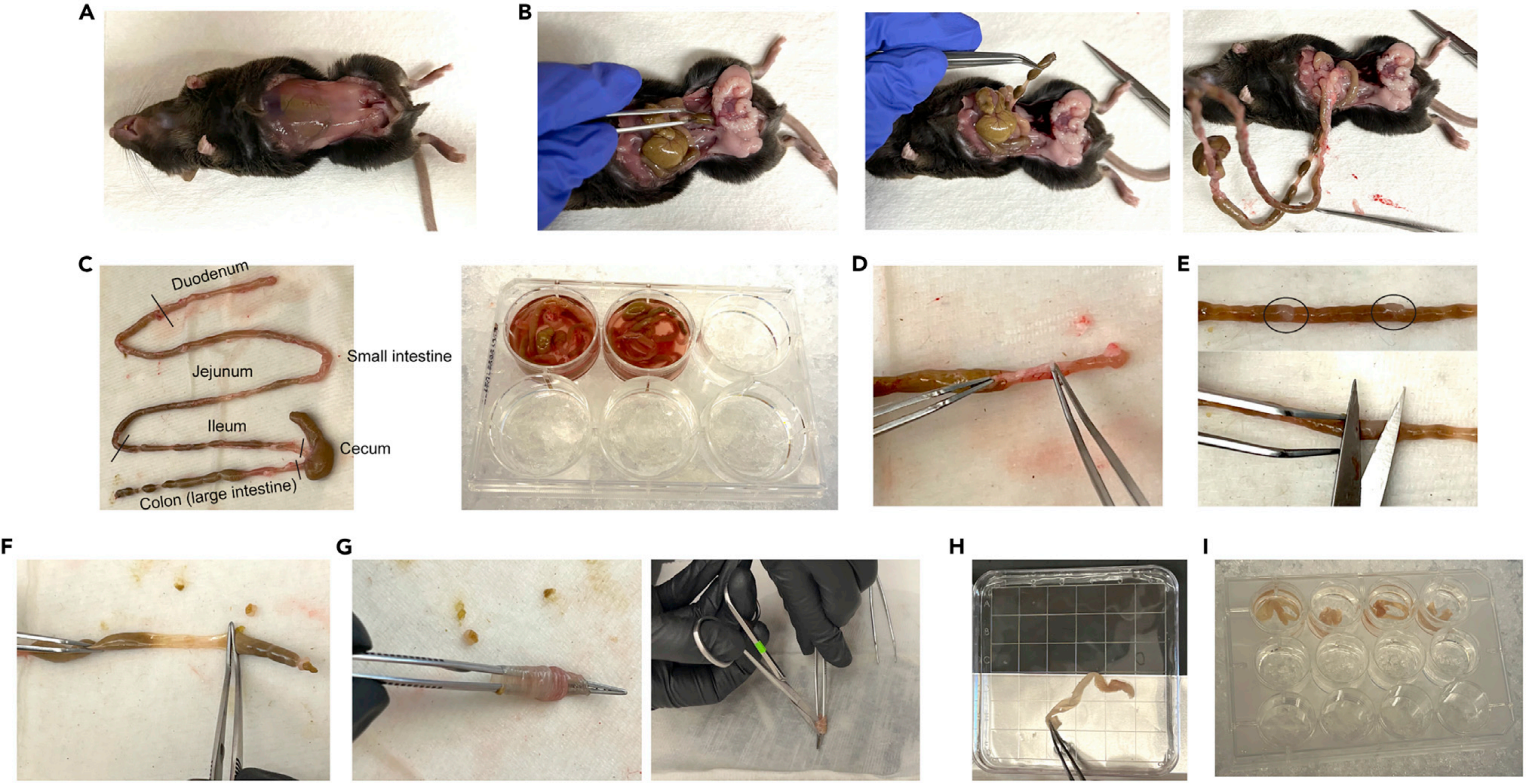
Processing the tissues (A) Expose the abdominal wall. (B) Cut the terminal colon and gently pull out the intestines. (C) Mouse intestinal anatomy. (D) Trim the surrounding fat tissues. (E) Remove Peyer’s patches. (F) Remove the intestinal content. (G) Cut the intestine longitudinally. (H) Rinse the tissue in ice-cold HBSS. (I) Transfer the tissue into a new well with 5 mL HBSS on ice.
  c. Find the terminal colon and cut it free with scissors. Then, gently pull out the intestines. (Figure 1B).
  d. Take the desired part of the intestines and place in a multi-well-plate with 5 mL of HBSS (w/o Ca^2+^, Mg^2+^) on ice, while other samples are being processed (Figure 1C).

1. Euthanize mice by CO_2_ inhalation or other means of euthanasia.
2. Collect intestines from the mice.
  a. Make a small incision in the abdomen.
  b. Holding each side of the incision, rip open the outer skin (Figure 1A).

**CRITICAL:** Keep all samples submerged in HBSS and on ice while processing other samples, as dried tissues will have reduced viable cell yield.

***Optional:*** Addition of 2% FBS into HBSS may improve cell viability.

### Trimming the tissue

**Timing: 5 min/ sample**

These steps prepare each sample for subsequent dissociation by removing tissues that can interfere with lymphocyte extraction and analysis.

3. Trim the intestine samples (Methods video S1).
  a. Transfer the intestines onto ice-cold HBSS soaked paper towels and trim the surrounding fat tissues (Figure 1D) and Peyer’s patches (Figure 1E).
  b. Remove the intestinal content thoroughly by gently pushing it from one end of the gut and out the other end (Figure 1F). Alternatively, a syringe can be used to inject HBSS inside of the intestines to remove the intestinal contents.
  c. Slide in angled forceps into the intestines and cut longitudinally (Figure 1G).
  d. Wash the sample twice by placing it into ice-cold HBSS and clean off mucus by gently scrubbing the tissue on the wet paper towel (Figure 1H).
  e. Transfer the tissue into a new multi-well plate with 5 mL HBSS on ice while other samples are being processed (Figure 1I).

**CRITICAL:** Ensure all Peyer’s patches are removed from the gut, as these may greatly impact immune cell composition results. Try to minimize the time that samples are off ice to keep cells alive. Incomplete removal of mucus or tissues drying out can also reduce the yield.

Video S1. Tissue trimming, related to step 3

### Removing epithelial cells and intraepithelial lymphocytes

**Timing: 0.5 h**

These steps dissociate unwanted cell populations from the intestinal tissues, including epithelial cells and intraepithelial lymphocytes, and leave primarily cells from the lamina propria.

**Figure 2:**
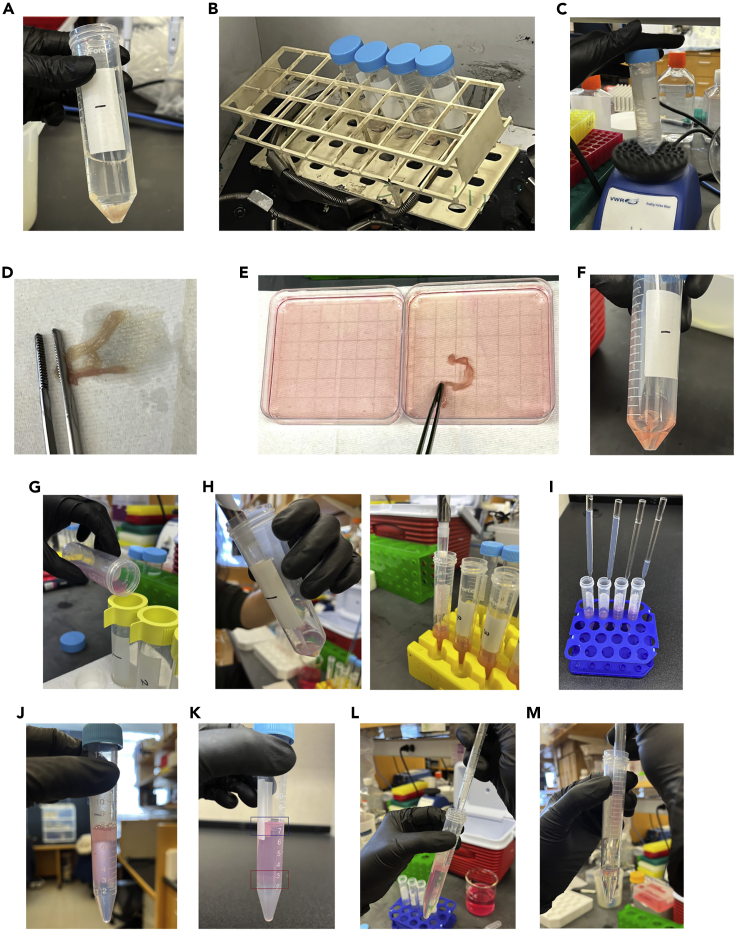
Isolating lamina propria lymphocytes (A) Transfer the intestinal tissues to the DTT-EDTA buffer. (B) Incubate the tissues in a shaker. (C) Vortex vigorously. (D) Remove residual solutions by scrubbing the tissues on a paper towel. (E) Rinse the tissues with ice-cold RPMI-1640 media. (F) Transfer the tissues to the digestion buffer. (G) Transfer the digested tissues to the strainer top tubes. (H) Resuspend the cell pellets with 1 mL of 40% Percoll solution and transfer to the prepared 15 mL conical tube with 4 mL of 40% Percoll solution. (I) Add 2.5 mL of 80% Percoll solution to the bottom of the tubes using glass Pasteur pipettes. (J) The LPLs from intestines before the centrifugation with Percoll. (K) The LPLs from intestines after the centrifugation with Percoll (red box). Blue box indicates epithelial cells. (L) Remove the upper 40% Percoll solution from the top. (M) Collect the cells (lymphocytes) from the interface.
6. Incubate the tissues in a shaker at 37°C, 250 rpm for 15 min (Figure 2B).
  a. Place the tubes at a 45° angle for better shaking.
7. Vortex each sample vigorously at maximum speed (∼3,000 rpm) for 10 s (Figure 2C).
8. Prepare 2 Petri dishes with 20 mL ice-cold RPMI-1640 media.
9. Take the tissue out of the EDTA-DTT buffer and remove residual solution by scrubbing the tissue on a clean paper towel (Figure 2D).
10. Rinse the tissues with ice-cold RPMI-1640 media (Figure 2E), then scrub on a clean paper towel. Repeat this step to thoroughly remove dissociated cells.

***Note:*** If epithelial cells or intraepithelial lymphocytes are the desired population for analysis, EDTA (for intestinal epithelial cells) and DTT (for intraepithelial lymphocytes) buffers can be treated separately and the supernatant saved after each reaction.

4. Prepare 15 mL of EDTA-DTT buffer per sample.
  a. Prewarm at 37°C until use.
  b. Prepare a 50 mL conical tube per sample.
  c. Aliquot 15 mL of EDTA-DTT buffer to each conical tube.
5. Transfer the intestinal tissues to the 50 mL conical tubes, ensuring tissues are submerged in EDTA-DTT buffer (Figure 2A).

### Isolation of lymphocytes from lamina propria

**Timing: 2–2.5 h**

These steps will generate a single cell suspension of the lamina propria cells, then isolate lymphocytes via a Percoll density gradient.

11. Prepare 5 mL digestion media per sample.
  a. Prewarm RPMI-1640 media at 37°C.
  b. Freshly prepare the digestion media with the prewarmed RPMI-1640 media before use.
    ***Note:*** The concentration of Liberase should be adjusted depending on the tissue types. See the materials and equipment section.
  c. Prepare a 50 mL conical tube per sample.
  d. Aliquot 5 mL of digestion buffer to each conical tube.

12. Transfer the tissues to the digestion buffer in the prepared conical tubes (Figure 2F).
13. Incubate the tissues in a shaker at 37°C, 250 rpm for 40 min to 1 h.
  a. Place the tubes at a 45° angle for better shaking.
  b. Vigorous shaking is recommended every 15 min for more efficient digestion.

***Note:*** Optimal digestion time can be varied depending on the tissue type, size, and disease-induced conditions. Usually, 35 min incubation for small intestines and 50 min for large intestines are optimal for efficient digestion while keeping high cell viability. For colitis-induced colons, longer incubation is recommended. See troubleshooting 1.

14. Prepare new 50 mL conical tubes with 100 μm cell strainers on the top.
15. Transfer the digested tissues through the strainers into the new tubes (Figure 2G).
  a. Vortex vigorously after incubation for 10 s.
  b. Pour the digested cell suspensions through the cell strainer. Small non-dissociated pieces of tissues can be discarded.
  c. Add 15 mL ice-cold HBSS to the original 50 mL conical tube to rinse out residual cells and pour through the cell strainer.
16. Centrifuge the samples at 450 × *g* for 10 min at 4°C.
17. Carefully remove the supernatant by vacuum suction or pouring out while taking care not to disturb the cell pellet.
18. Prepare 80% Percoll and 40% Percoll solutions, then prepare 40% Percoll tubes per sample.
  a. Prepare a 15 mL conical tube for each sample.
  b. Aliquot 4 mL of 40% Percoll solution to each conical tube.
19. Resuspend the cell pellets with 1 mL of 40% Percoll solution and transfer to the prepared 15 mL conical tube with 4 mL of 40% Percoll solution (Figure 2H and Methods video S2).
20. Add 2.5 mL of 80% Percoll solution to the bottom of the 15 mL tubes (Figure 2I and Methods video S2).
  a. Place a glass Pasteur pipette into each tube with the 40% Percoll cell suspension, making sure the pipette reaches the bottom of the tube.
  b. Slowly apply 2.5 mL of 80% Percoll solution through the glass Pasteur pipette to the bottom of the tube.
  c. Wait until the 80% Percoll solution is completely added before removing the glass Pasteur pipettes to leave the 40/80% Percoll interface intact.
21. Gently move the samples to the centrifuge, while not disturbing the 40/80% Percoll interface (Figure 2J).
22. Centrifuge the samples at 860 × *g* for 20 min at 21°C with the lowest acceleration speed (acceleration 0 or 1) and no brake (deceleration 0).

**CRITICAL:** Lowering acceleration and deceleration speed help create a clearer interface to identify lymphocytes. Centrifugation at 21°C is critical, as the density of Percoll changes at different temperatures.

23. Prepare a new 15 mL conical tube per sample and add 10 mL of HBSS.
24. Take the cells located at the interface of 40/80% Percoll solution (Figure 2K and Methods video S3). Troubleshooting 2.
  a. Remove the upper 40% Percoll solution from the top until ∼4 mL of total solution remain (Figure 2L).
  b. Use a transfer pipette to collect the cells (lymphocytes) at the interface (Figure 2M).
  c. Transfer the lymphocytes to the newly prepared 15 mL tube in step 23.
25. Thoroughly mix by inverting and centrifuge at 450 × *g* for 10 min at 4°C.
26. Discard supernatant.
27. Add 10 mL of HBSS to the pellet and centrifuge at 450 × *g* for 10 min at 4°C to wash the pellet. Resuspension is not required.
28. Discard supernatant.

**CRITICAL:** Complete Percoll removal is critical for cell viability, particularly in cases where cell stimulation is required. A second wash with HBSS after step 28 is optional to fully remove Percoll from the samples.

**Pause point:** The experiment can be paused at this point up to 12–16 h. To pause at this point, resuspend the cell pellet with 5 mL FACS buffer and keep the cells at 4°C.

***Note:*** If cell stimulation is not required for the analyses, the next section “Stimulation of lymphocytes” can be skipped. Resuspend the pellet with 200 μL HBSS and transfer the cells to the 96-well round bottom plate and directly proceed for “Staining for flow cytometry analyses”.

Video S2. 80% Percoll seeding, related to steps 19 and 20

Video S3. Retrieving lymphocytes from the Percoll interface, related to step 24

### Stimulation of lymphocytes

**Timing: 2.5–3 h**

If lymphocyte stimulation is required, these steps will activate the isolated lymphocytes for cytokine expression analyses.

29. Prewarm the 2× T cell stimulation media and aliquot 100 μL into a 96-well round bottom plate for each sample, plus one well for the unstained control.
30. Resuspend each sample cell pellet from step 28 with base T cell culture media.

***Note:*** The volume of media for the resuspension can be adjusted based on the size of the cell pellet. 1 × 10^6^ to 2 × 10^6^ cells are recommended for the stimulation. Count the cell number using a Burker chamber or automated cell counter with Trypan Blue staining for live/dead cell discrimination for the representative sample and determine the total volume of the resuspension. e.g., If 3 × 10^6^ cells are harvested and 1 × 10^6^ are desired for stimulation, resuspend the cell pellets with 300 μL of the base T cell culture media. If the number of cells in a specific cell population needs to be calculated, please remember the resuspension volume in case you need to calculate back the cell numbers after flow cytometry analyses. Troubleshooting 3.

31. Take 100 μL of each sample cell suspension and add it to the corresponding well containing 2× T cell stimulation media.
32. Thoroughly mix by pipetting gently.
33. Take 100 μL of any residual cell suspension and add it to the unstained control well.

***Optional:*** If fluorescence minus one staining (FMO) controls are required (Tung et al., 2007), save 100 μL of any residual cell suspension into additional wells.

34. Incubate the 96-well plate in a 5% CO_2_, 37°C cell culture incubator for 2–3 h.

### Surface markers staining for flow cytometry analyses

**Timing: 0.5 h**

These steps will stain the obtained lymphocytes with antibodies for surface marker proteins.

35. Take the 96-well plate from the cell culture incubator.
36. Centrifuge the 96-well plate at 450 × *g* for 2 min at 4°C.
37. Remove the supernatant by flipping the 96 well plate.
38. Wash cells by adding 200 μL/well of HBSS without resuspending. Repeat steps 36–38 twice.
39. Prepare fixable live/dead viability staining solution.

***Note:*** When preparing master mixtures used for staining in flow cytometry analyses, it may be helpful to lower the volume of diluent to account for any supernatant remaining in wells. For instance, if there is an estimated 15 μL of fluid left in wells, the volume of master mix can be reduced to 35 μL while keeping the total amount of reagent the same.

40. After the last centrifugation, remove the supernatant by flipping the 96-well plate.
41. Add 50 μL/well of fixable live/dead viability staining solution and resuspend.
42. Incubate at 4°C for 10 min. The plate should be covered with aluminum foil from this step on.
43. Add 150 μL/well of HBSS to wash and centrifuge at 450 × *g* for 2 min at 4°C.
44. Remove the supernatant by flipping the 96-well plate.
45. Add 50 μL/well of Fc Blocker solution and resuspend.

***Note:*** This step can be combined with antibody staining by creating a single master mix with both components. This has demonstrated comparable results to keeping these mixtures separate, but the efficacy may differ depending on antibodies.

46. Add 150 μL/well of HBSS to wash and centrifuge at 450 × *g* for 2 min at 4°C.
47. Remove the supernatant by flipping the 96-well plate.
48. Prepare antibody mix 1 as indicated in Table 1 for surface markers staining (Surface markers staining panel).
49. Add 50 μL/well of antibody mixture and resuspend cells gently.
50. Incubate at 4°C for 30 min. The plate should be covered with aluminum foil.
51. Add 150 μL/well of HBSS to wash and centrifuge at 450 × *g* for 2 min.
52. Remove the supernatant by flipping the 96-well plate.
53. Repeat steps 51 and 52.

***Note:*** If intracellular staining is unnecessary, skip steps 54–65 and proceed to step 66.

### Intracellular cytokines and transcription factors staining for flow cytometry analyses

**Timing: 1.5 h**

These steps will fix and permeabilize the lymphocytes and stain with antibodies for intracellular cytokines and transcription factors.

54. Prepare Foxp3 Fixation/Permeabilization working solution.
55. Resuspend the cells with 150 μL/well of the working solution and incubate for 20–30 min at 20°C–22°C. The plate should be covered with aluminum foil.

**Pause point:** The experiment can be paused at this point with a 12–16 h incubation at 4°C. Otherwise, proceed after incubating for 20–30 min at 20°C–22°C.

56. Centrifuge at 860 × *g* for 2 min and remove supernatant by flipping the plate. Troubleshooting 4.
57. Prepare 1× Permeabilization buffer working solution.
58. Add 200 μL of Permeabilization buffer to each well.
59. Centrifuge at 860 × *g* for 2 min and remove supernatant by flipping the plate.
60. Repeat steps 58 and 59.
61. Prepare the antibody mix as indicated in Table 2 for intracellular cytokines and transcription factors (Intracellular cytokine/transcription factors staining panel).
62. Add 50 μL of antibody mixture to each well.
63. Incubate at 4°C for 1 h. The plate should be covered with aluminum foil.
64. Add 150 μL of 1× Permeabilization buffer working solution and centrifuge at 860 × *g* for 2 min, then remove supernatant by flipping the plate. Repeat twice.
65. Resuspend the cells with 200 μL of FACS buffer.

***Optional:*** If cell numbers need to be counted, add counting beads (Cat. 424902, Biolegend) to each well according to the manufacturer’s protocol (https://www.biolegend.com/en-ie/products/precision-count-beads-13279). Cell numbers can be calculated back after flow cytometry runs.

### Data collection

66. Collect data with flow cytometry analyzer (LSRII, BD Biosciences) and analyze by using FlowJo software (FlowJo, LLC). Prior to running the samples, appropriate PMT voltage and compensation are required. The usage of compensation beads with the same set of antibodies from each panel is recommended. Compensation control for Fixable live/dead aqua can be prepared with amine-reactive compensation bead kit. Troubleshooting 5 and 6.

## Expected outcomes

This protocol outlines the purification of live lymphocyte populations from the mouse lamina propria for analysis of gut mucosal immune cell populations and their perturbations. Using this protocol, we expect to obtain up to 80% of viable intestinal lamina propria lymphocytes, and can define B cells (AmCyan^-^CD45^+^TCRβ^-^CD3ε^-^CD19^+^), non-αβ T cells (likely γδ T cells, AmCyan^-^CD45^+^TCRβ^-^CD3ε^+^), CD8^+^ T cells (AmCyan^-^CD45^+^TCRβ^+^CD3ε^+^CD19^-^CD4^-^CD8^+^), CD4^+^ T cells (AmCyan^-^CD45^+^TCRβ^+^CD3ε^+^CD19^-^CD8^-^CD4^+^), and subsets of CD4^+^ T cells such as IL-17A-, IFNγ-, RORγt-, FoxP3-, RORγt and FoxP3-expressing T cells (Th1, Th17, and regulatory T cells). With the range of cell types that can be detected, this protocol enables the detailed study of broad intestinal immune cell composition and how different treatments or states affect the differentiation of specific immune cell types as well as overall gut immune tone.

**Figure 3:**
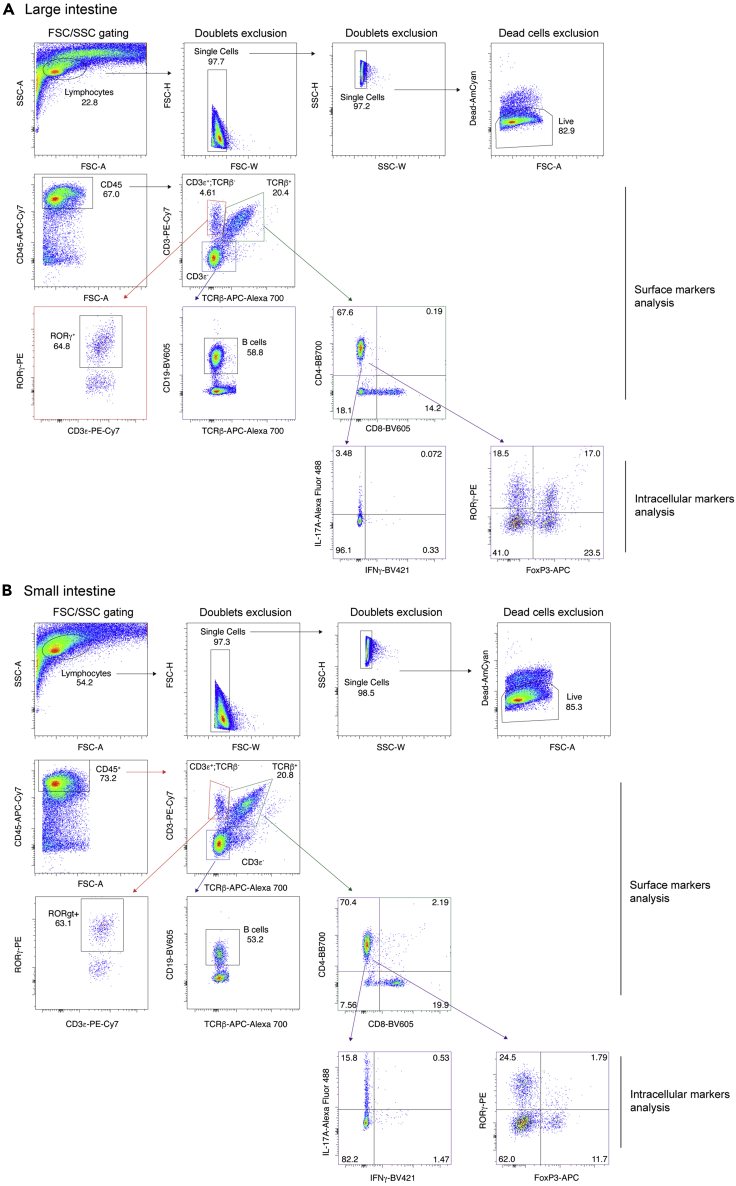
Gating strategy for T cells and expected results (A and B) The gating strategy of B cells, non-αβ T cells, CD8^+^ T cells, CD4^+^ T cells and its subpopulations. First, roughly gate on lymphocytes based on FSC and SSC, and exclude doublets by FSC-A/FSC-H and SSC-A/SSC-H. Then, exclude dead cells by gating on the AmCyan-low population. Next, gate on the CD45-positive cells for pan-leukocytes, then gate on CD3ε and TCRβ to differentiate αβ T cells from non-T cells and non-αβ T cells (γδ T cells). By gating on CD3ε^+^ TCRβ^-^ cells, RORγ-expressing γδ T cells can also be defined. Among non-T cells, B cells can be defined by gating on the CD19-positive population. After gating on αβ T cells, either CD4^+^ or CD8^+^ T cells can be determined. Lastly, among CD4-positive T cells, IL-17A^+^ IFNγ^+^ cells and RORγ^+^ FoxP3^+^ cells can be defined. Potential results from colon (A) and ileum (B). **Note:** We used the same fluorophore for CD8a and CD19 because we can differentiate CD8 T cells by gating on TCRβ, CD3ε-positive and CD8a (BV605), and B cells on TCRβ, CD3ε-negative, and CD19-positive (BV605).

Our protocol can be used to characterize the immune cell compositions of different tissue types, such as the large (Figure 3A) and small intestines (Figure 3B) which are immunologically distinct within the same individual. The composition of immune cells in the intestinal lamina propria is also greatly affected by vivarium. For example, in mice that are colonized with segmented filamentous bacteria (SFB), almost 15–20% of all CD4^+^ T cells in the small intestine (particularly ileum) are IL-17A-producing Th17 cells (Ivanov et al., 2009) (Figure 3B), while in SFB-negative mice this proportion is less than 5%. These observations underscore the importance of including proper control groups during experimental design, but also highlight the sensitivity of this protocol in detecting the impact of small-scale changes.

## Limitations

The antibody panels that we used in this protocol are not sufficient to analyze all lymphocytes population in the intestinal lamina propria, such as ILCs. For analyzing lymphocyte populations other than T cells, antibody panels for surface, intracellular, and transcription factor staining can be modified (Wang et al., 2020). Additionally, as noted earlier, intestinal immune cell composition can be highly sensitive to a variety of factors, including housing conditions and microbial exposures. This may greatly affect results if the desired immune cell populations are over-/under-induced at baseline due to such confounding factors, so it is imperative that experiments are controlled accordingly.

## Troubleshooting

### Problem 1

Tissues are not completely digested (step 13).

### Potential solution

Intestinal tissues from inflammation-induced conditions (e.g., colitis) can be thicker and tougher compared to the homeostatic condition. Increasing the frequency of vigorous shaking during the digestion or 10–15% extension of digestion time can be helpful up to 1 h. Chopping the tissues prior to digestion may also help, but is not required. Excessive mechanical dissociation may lower the cell viability.

### Problem 2

Few to no cells are visible at the 40/80% Percoll interface (step 24).

### Potential solution

When trying to analyze the lamina propria lymphocytes from a specific location of small intestinal tissue (e.g., duodenum or terminal ileum) in homeostatic condition, cells may not be clearly visible at 40/80% Percoll interface. Even if the interface is faint, we recommend proceeding with the protocol.

If too many fat tissues remain after tissue trimming, or mucus is not completely removed after the EDTA-DTT buffer incubation steps, lymphocytes may become trapped in these tissues which will prevent the pelleting of lymphocytes from the single-cell suspension.

### Problem 3

Low cell yield (step 30).

### Potential solution

Flow cytometry results can still be reliable with a small number of cells. Even if the cell yield does not reach optimal numbers, cell stimulation can still proceed with the same conditions.

To increase cell yield, make sure to extract as many of the cells at the 40/80% Percoll interface as possible while avoiding disturbing the 80% layer. Thorough removal of fat and mucus during trimming and digestion steps may also help to increase cell counts. Care when pipetting cells and removing supernatant, especially at small volumes, will also raise final cell yields.

### Problem 4

Cell pellets are undetectable (step 56).

### Potential solution

After the fixation and permeabilization step, it is expected that the cell pellets become less visible, particularly in the case that the starting numbers of cells were small. To avoid the loss of fixed/permeabilized cells, we typically increase the speed of centrifugation to 860 × *g* after cells have been fixed (step 56). We also recommend moving forward with the protocol, as we have found that samples with undetectable cell pellets still often yield meaningful cell counts.

### Problem 5

Low cell viability (step 66).

### Potential solution

Optimal usage of Liberase concentration and digestion time are critical to cell viability. To immediately stop the digestion reaction immediate addition of 10 mL ice-cold HBSS to the digestion buffer is helpful. Complete removal of Percoll before stimulating the cells with stimulation media can be also critical. Residual Percoll can be confirmed under the microscope.

In addition, we have observed that exposure to specific microbes can impede the isolation of viable immune cells from the lamina propria. Finely controlling housing conditions such as maintaining cages independently to limit cross-exposure from handling other mice can improve yield if this is an issue.

### Problem 6

Antibody staining of cell markers is ineffective (step 66).

### Potential solution

We recommend designing antibody panels such that the brightest antibodies are used for targets with the lowest expression levels. For instance, in our antibody panel, we have used PE and APC for weak targets (RORγt and FoxP3) which we found improved their detection. There are several online tools that may assist in optimizing antibody panel design (https://www.bdbiosciences.com/content/dam/bdb/marketing-documents/Fluorochrome-Chart-Relative-Brightness.pdf).

## Resource availability

### Lead contact

Further information and requests for resources and reagents should be directed to and will be fulfilled by the lead contact, Jun R. Huh (Jun_Huh@hms.harvard.edu).

### Materials availability

This study did not generate new unique reagents.

## Data Availability

This study did not generate or analyze any datasets.
